# Biologic activity of the novel orally bioavailable selective inhibitor of nuclear export (SINE) KPT-335 against canine melanoma cell lines

**DOI:** 10.1186/1746-6148-10-160

**Published:** 2014-07-15

**Authors:** Megan N Breit, William C Kisseberth, Misty D Bear, Yosef Landesman, Trinayan Kashyap, Dilara McCauley, Michael G Kauffman, Sharon Shacham, Cheryl A London

**Affiliations:** 1 Departments of Veterinary Biosciences and Veterinary Clinical Sciences, College of Veterinary Medicine, The Ohio State University, 1925 Coffey Rd., Columbus, OH 43210, USA; 2 Karyopharm Therapeutics, Natick, MA, USA

**Keywords:** XPO1, Malignant melanoma, Dog

## Abstract

**Background:**

Exportin 1 (XPO1, also known as CRM1), is a chaperone protein responsible for the export of over 200 target proteins out of the nucleus. The expression and activity of XPO1 is upregulated in several human cancers and its expression is also linked to the development of chemotherapy resistance. Recent studies using both human and murine cancer cell lines have demonstrated that XPO1 is a relevant target for therapeutic intervention. The present study sought to characterize the biologic activity of an orally bioavailable selective inhibitor of nuclear export (SINE), KPT-335, against canine melanoma cell lines as a prelude to future clinical trials in dogs with melanoma.

**Results:**

We evaluated the effects of KPT-335 on 4 canine malignant melanoma cell lines and found that KPT-335 inhibited proliferation, blocked colony formation, and induced apoptosis of treated cells at biologically relevant concentrations of drug. Additionally, KPT-335 downregulated XPO1 protein while inducing a concomitant increase in XPO1 messenger RNA. Lastly, KPT-335 treatment of cell lines upregulated the expression of both protein and mRNA for the tumor suppressor proteins p53 and p21, and promoted their nuclear localization.

**Conclusions:**

KPT-335 demonstrates biologic activity against canine melanoma cell lines at physiologically relevant doses, suggesting that KPT-335 may represent a viable treatment option for dogs with malignant melanoma.

## Background

Canine oral malignant melanoma is a biologically aggressive tumor with a reported metastatic rate of up to 90% [1]. Local disease can be treated with surgery and coarse-fractionated radiation therapy, with success dependent on location and stage [1]. Despite options for local control most dogs will eventually die of metastatic disease [2]. Similar to the case with human malignant melanoma, the use of traditional chemotherapy agents, such carboplatin and cisplatin, has little impact on both the primary tumor and metastatic disease, with no substantial survival benefit [3-7]. Other treatment strategies, such as immunotherapy using a variety of vaccine based approaches, including the commercially available xenogeneic melanoma vaccine (Oncept), have been explored as options for the management of canine malignant melanoma [8-10]. While the Oncept vaccine was proven to be safe and preliminary data indicated that it significantly enhanced survival when used in the setting of loco-regional control, [11] a recent retrospective clinical study failed to replicate these findings dogs with oral melanoma that had loco-regional tumor control [10-14]. There is also limited data in which small molecule inhibitors such as toceranib phosphate (Palladia) have been used to treat this disease in dogs, although formal data regarding efficacy is lacking [15,16]. Therefore, new therapeutic approaches to treatment are needed.

Recently, the protein exportin 1 (XPO1, also called Chromosome Region Maintenance protein 1 [CRM1]) has been validated as a target for therapeutic intervention in cancer. XPO1 is one of seven known nuclear export proteins responsible for shuttling cargo from the nucleus to the cytoplasm [17-19]. It is a member of the karyopherin β family of transport receptors that binds over 200 target proteins through a hydrophobic leucine-rich nuclear export signal (NES) present in the cargo [20]. XPO1 is the sole nuclear exporter of several major tumor suppressor and growth regulatory proteins (TSPs and GRPs), including p53, p75, Rb, p21, p27, STAT3, FOXO and IκB among others [21,22]. There is now substantial data demonstrating that XPO1 is upregulated in both hematologic malignancies and solid tumors [17-19]. Furthermore, overexpression of XPO1 correlates with a poor prognosis in many human cancers, indicating that changes in nuclear-cytoplasmic trafficking resulting in aberrant localization of key proteins can contribute to cancer development and progression.

Given the role of XPO1 dysregulation in cancer, there has been great interest in developing inhibitors of this protein. Recently, novel orally bioavailable, small-molecule selective inhibitor of nuclear export (SINE) compounds that specifically bind to XPO1 at the reactive site Cys 528 residue have been developed and tested both *in vitro* and *in vivo*[23-29]. SINE compounds induce apoptosis and block proliferation in several cancer cell lines, including those derived from colon [21], pancreas [23], and breast carcinomas [27] as well as chronic lymphocytic leukemia (CLL) [26], while sparing normal cells [30]. Additional studies have shown potent anti-cancer activity and good tolerability of SINE *in vivo* using mouse human xenograft (subcutaneous, orthotopic, or leukemograft) models of pancreatic cancer [23], renal cancer [31], CLL [26], mantle cell lymphoma (MCL) [29], multiple myeloma [32] and acute myelogenous leukemia (AML) [28]. Early clinical trials of the SINE KPT-330 (selinexor) have demonstrated biologic activity of XPO1 inhibition in human lymphoid malignancies.

The SINE compound KPT-335 (verdinexor, closely related to selinexor) has been previously evaluated in canine lymphoma cell lines and found to have good activity in the low nanomolar range [33]. Additionally, a phase I clinical trial of KPT-335 in dogs with primarily lymphoma demonstrated evidence of single agent activity consisting of both partial response to therapy and stable disease for over 4 weeks with excellent tolerability over long-term dosing. Lastly, data generated in both healthy dogs and dogs with cancer indicate that KPT-335 exhibits good oral bioavailability with an average Cmax of approximately 250 ng/ml and an average AUC of 1800 ng/ml [33]. The purpose of this study was to evaluate the activity of KPT-335 against established canine malignant melanoma cell lines as a prelude to future testing in dogs with metastatic melanoma.

## Methods

### Cell lines and reagents

Canine melanoma cell lines Mel 23, Mel 36, Mel 69 and Mel 83 were generously provided by Michael S. Kent (UC Davis School of Veterinary Medicine, Davis, CA) [34-36]. Three of the lines (Mel 23, 69 and 83) were derived from a primary oral tumor and Mel 36 was generated from a metastatic lymph node. The cell lines were maintained in RPMI 1640 supplemented with 10% FBS, non-essential amino acids, sodium pyruvate, penicillin, streptomycin, L-glutamine, and Hepes (4-(2-hydroxythyl)-1-piperazineethanesolfonic acid) at 35°C, supplemented with 5% CO₂. KPT-335 (provided by Karyopharm Therapeutics, Inc, Natick, MA) was dissolved in DMSO to generate stock solutions for use *in vitro*. The following antibodies were used for Western blotting and immunofluorescence experiments: anti-p53, anti-p21, anti-XPO1, and anti- β-actin (all from Santa Cruz Biotechnology, Santa Cruz, CA).

### Cell proliferation assay

Melanoma cells (1.5 × 10^3^) were seeded in triplicate in 96-well plates overnight in 10% FBS supplemented media and incubated with DMSO or increasing concentrations of KPT-335. After 72 or 96 hours, the medium was removed and the plates were frozen at −80°C overnight before processing using the CyQuant® Cell Proliferation Assay Kit (Molecular Probes, Eugene, OR) according to the manufacturer’s instructions. Cell proliferation was calculated as a percentage of the DMSO-treated control wells and IC₅₀ values were derived after plotting proliferation values on a logarithmic curve. Each experiment was repeated 3 times.

### Assessment of apoptosis

The ability of KPT-335 to induce apoptosis in treated cells was assessed using annexin V/propidium iodide (PI) staining as previously described [37]. Briefly, 1.0 × 10^6^ canine melanoma cells were treated with 0.1% DMSO, 0.1 μM KPT-335, or 1 μM KPT-335 for 96 hours at 37°C. Cells were collected, washed, and stained with annexin V–fluorescein isothiocyanate and PI for 15 minutes before evaluation by flow cytometry on a Becton Dickinson FACS Caliber. Each experiment was repeated 3 times.

### Immunoblotting

Melanoma cells (2 × 10^6^) in 10% FBS medium were treated with DMSO, 0.1 μM or 1 μM KPT-335 for 4 or 24 hours. Protein lysates were prepared and quantified, separated by SDS-PAGE, and Western blotting was performed using previously described methods [38]. The membranes were incubated overnight with anti-XPO1, anti-p53 or anti-p21 antibodies, then incubated with the appropriate horseradish peroxidase linked secondary antibodies, washed, and exposed to substrate (SuperSignal West Dura Extended Duration Substrate, Pierce, Rockford, IL). Blots were stripped, washed, and reprobed for β-actin.

### Quantitative RT-PCR

Total RNA was extracted from canine melanoma cells that were cultured in 10% FBS supplemented medium for 4 or 24 hours with DMSO*,* 0.1 μM or 1 μM KPT-335, using TRIzol (Invitrogen). cDNA was made from 2 μg of total RNA using Superscript III (Invitrogen), followed by real-time PCR with TaqMan-specific probes (Applied Biosystems) according to the manufacturer’s protocol. Real-time PCR for XPO1 was performed using the Applied Biosystems StepOne Plus Detection System and MIC-1 and p21 expression was detected using the ViiA™ 7 Real-Time PCR System (Life Technologies). Normalization was performed relative to 18S rRNA. All reactions were performed in triplicate and included no-template controls for each gene. Relative gene expression for all real-time PCR data was calculated using the comparative threshold cycle method [39].

### Immunofluorescence

Cells were plated in a 24 well plate with poly-lysine coated coverslips (35,000-50,000 cells per well) then treated with DMSO or 1 μM KPT-335. They were then fixed with 4% paraformaldehyde and permeabilized with 0.2% Triton-X. Next, the cells were blocked at room temperature in blocking buffer (1x PBS/5% bovine albumin/0.3% Triton-x) for 30 minutes and then were incubated with anti-p53 or anti-p21 for 1 hour at room temperature. A secondary FITC labeled anti-rabbit or anti-goat antibody was applied for 30 minutes, as appropriate (Alexa Fluor® 488 goat anti-rabbit IgG Invitrogen or Alexa Fluor® 488 donkey anti-goat IgG Invitrogen). Cells were also stained with DAPI to visualize the nucleus (ProLong® Gold antifade reagent with DAPI Invitrogen). Intracellular localization of proteins was analyzed by immunofluorescence microscopy using an Olympus FV1000 Spectral confocal microscope.

### Clonogenic assay

Melanoma cell lines were grown in flasks until 80% confluent, then collected, washed and plated at 2,000 cells per well in six-well plates. After 24 hours the cells were treated with DMSO, 1 nM, 10nM, 0.1 μM, 1 μM or 10 μM KPT 335 and incubated at 35°C, supplemented with 5% CO₂ for 7 days. Cells were then fixed with methanol/acetic acid (3:1), washed with PBS and stained with crystal violet (0.5%). The surviving cell fraction was defined as the number of colonies counted divided by the number of cells that were plated in the treated groups and then normalized to the plating efficiency. Plating efficiency was defined as the number of colonies divided by the number of cells plated in the untreated group [34,35]. Vehicle control treated Mel 23 and 36 cells served as the plating efficiency control group. Experiments were performed in triplicate.

### Statistics

All experiments were performed 3 times and/or performed in triplicate. A mean and standard deviation was derived from all repeated experiments. The apoptosis assays and clonogenic assays were evaluated using a Student’s t-test to compare treated groups to vehicle control. A one-way ANOVA comparison test was used to evaluate differences in gene expression among the KPT-335 treated and vehicle control treated groups for the quantitative RT-PCR assays and to compare multiple treatment groups in the cell proliferation assays. Values of p < 0.05 were considered statistically significant.

### Ethical support

All the studies we performed were in vitro with cell lines and as such, no IACUC or approval was necessary. The cell lines have been available for several years and have been used in prior publications.

## Results

### KPT-335 inhibits the proliferation of canine melanoma cell lines

**Figure 1:**
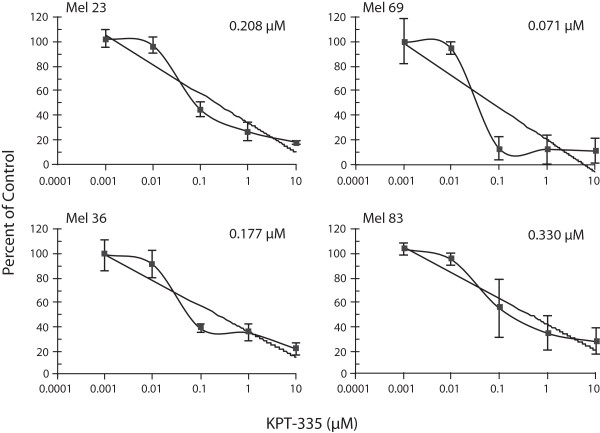
**KPT-335 inhibits proliferation of canine melanoma cells.** Canine melanoma cell lines were treated with DMSO or KPT-335 at increasing concentrations for 96 hours then analyzed using the CyQUANT® cell proliferation kit. Proliferation values are listed as a percentage of DMSO control. Experiments were performed in triplicate and repeated three times. For each cell line there was a significant decreasing trend in cell proliferation with dose of KPT-335 (p < 0.0001).

To assess whether KPT-335 was capable of inhibiting the proliferation of canine melanoma cells, the tumor cell lines were treated with increasing concentrations of KPT-335 and the CyQUANT assay was used to assess relative cell numbers after 96 hours of culture. As demonstrated in Figure 1, proliferation of all cell lines was significantly inhibited at concentrations in the nanomolar range. The IC₅₀ values for the various cell lines ranged from 0.071-0.330 μM.

### XPO1 inhibition induces apoptosis of canine melanoma cell lines

**Figure 2:**
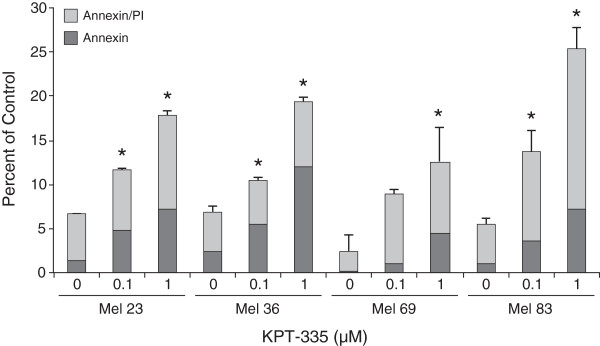
**KPT-335 induces apoptosis of canine melanoma cells.** Canine melanoma cell lines were treated with DMSO, 0.1 μM KPT-335 or 1 μM KPT-335 for 96 hours. Cells were collected and stained with Annexin V FITC/propidium iodide and analyzed by flow cytometry to quantitate early and late apoptosis. Cells treated with KPT-335 at 0.1 μM and 1 μM demonstrated a substantial increase in early and late apoptosis compared to vehicle treated cells (p < 0.05; with the exception of the Mel 69 in which only the 1 μM late apoptosis group showed statistical significance).

To determine if inhibition of XPO1 resulted in apoptosis of canine melanoma cell lines, cells were treated with DMSO, 0.1 μM KPT-335 or 1 μM KPT-335 for 96 hours. Following collection, they were stained with markers of early (AnnexinV) and late (propidium iodide) apoptosis and then analyzed by flow cytometry. Figure 2 demonstrates that treated cells exhibited significant increases in early and late apoptosis when compare to the vehicle control in the 0.1 μM and 1 μM treatment group, with the exception of Mel 69 which only had a significant increase in late apoptosis in the 1 μM treatment group.

### Colony formation is inhibited by KPT-335

**Figure 3:**
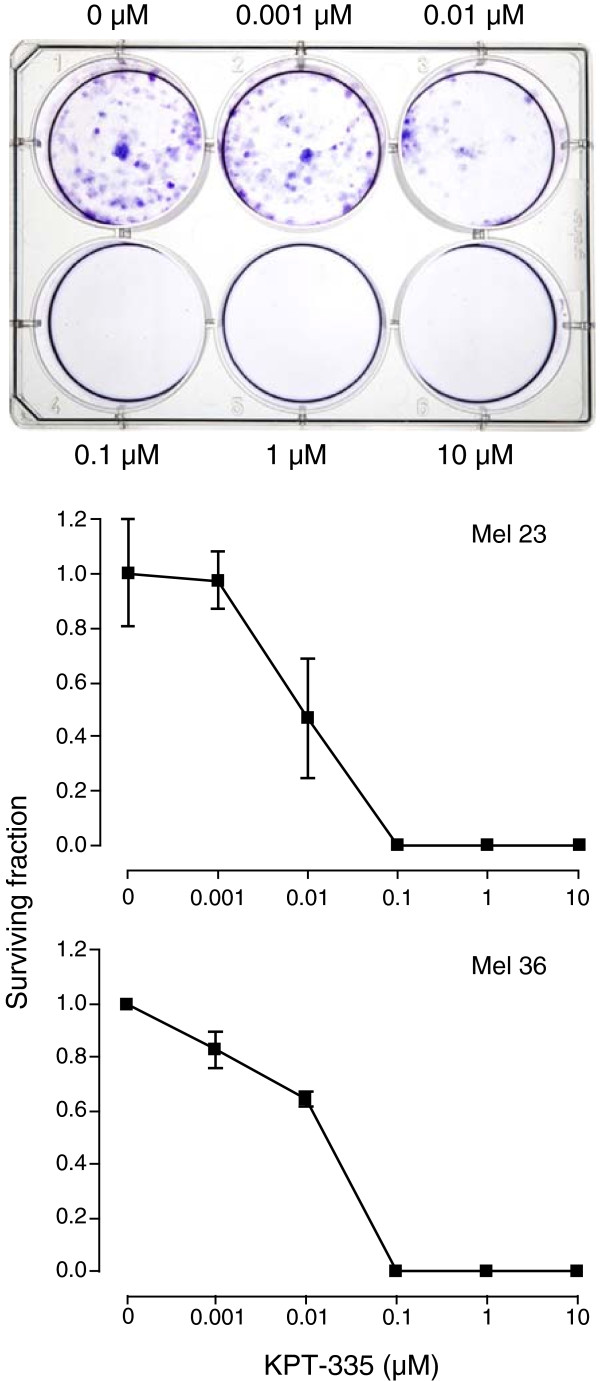
**Effect of KPT-335 on colony formation.** Canine Mel 23 and Mel 36 cells were seeded at 2,000 cells per well in a 6 -well plate for 24 hours, followed by treatment with DMSO, 0.001, 0.01, 0.1, 1 or 10 μM KPT-335 for 7 days. Cells were then fixed and stained with crystal violet and colonies with greater than 50 cells were counted. The surviving cell fraction was defined as the number of colonies counted divided by the number of cells that were plated in the treated groups and then normalized to the plating efficiency. Plating efficiency was defined as the number of colonies divided by the number of cells plated in the untreated group. Vehicle control treated Mel 23 and Mel 36 cells served as the plating efficiency control group. The surviving fraction was significantly decreased in cells treated with > 10 nM concentration of KPT-335, p < 0.05.

Mel 36 and Mel 23 previously have been demonstrated to exhibit colony formation in culture and inhibition of colony growth has been used as a tool to assess the effects of various treatments including rapamycin, nutilin-3 and DNA damaging agents on these canine melanoma cell lines [34,35]. To determine whether blocking XPO1 function would impair the formation of colonies, Mel 23 and Mel 36 cells were seeded in 6-well plates and treated with DMSO or KPT-335 and evaluated daily. After 7 days of culture the plates were collected and colonies were counted following staining with crystal violet. A significant inhibition of colony formation occurred in cells exposed to > 10 nM of drug when compared to vehicle control (Figure 3).

### KPT-335 downregulates XPO1 protein expression while inducing a concomitant increase in XPO1 mRNA

**Figure 4:**
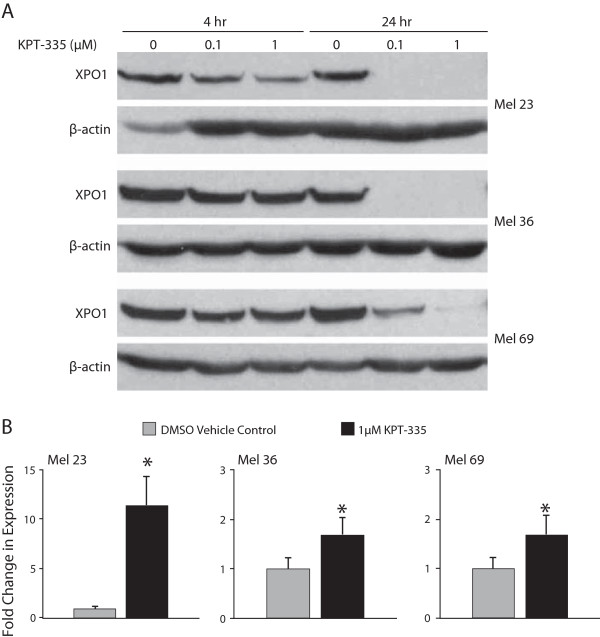
**Impact of KPT-335 on XPO1 message and protein expression in melanoma cell lines. A**. Canine melanoma cell lines were treated with DMSO or KPT-335 1 μM for 24 hours and RNA was collected. Quantitative RT-PCR was performed for XPO1. Relative expression was found to be increased in treated cell lines with a p< 0.05. **B**. Canine melanoma cell lines were treated with DMSO or KPT-335 at 0.1 μM or 1 μM for 4 or 24 hours prior to collection. Protein lysates were generated, separated by SDS-PAGE and Western blotting for XPO1 and β-actin were performed.

Previous work has shown that XPO1 inhibition by SINE compounds results in loss of target protein, while simultaneously inducing upregulation of XPO1 gene expression [32]. Furthermore, increased expression of XPO1 mRNA has been used as a pharmacodynamic marker in people treated with the closely related SINE compound KPT-330 (selinexor) [40]. To determine whether KPT-335 has a similar effect on XPO1 in canine melanoma cell lines, cells were incubated with DMSO or KPT-335 at 0.1 μM or 1 μM for 4 and 24 hours. Figure 4A demonstrates a time and dose dependent loss of protein expression in treated cells. Concordant with results in human cell lines, XPO1 mRNA was similarly upregulated in the melanoma cells following incubation with KPT-335 for 24 hours (Figure 4B), demonstrating a compensatory response to loss of XPO1 protein.

### KPT-335 modulates the expression of p53 and its downstream targets and localization of p53 and p21 in canine melanoma cell lines

**Figure 5:**
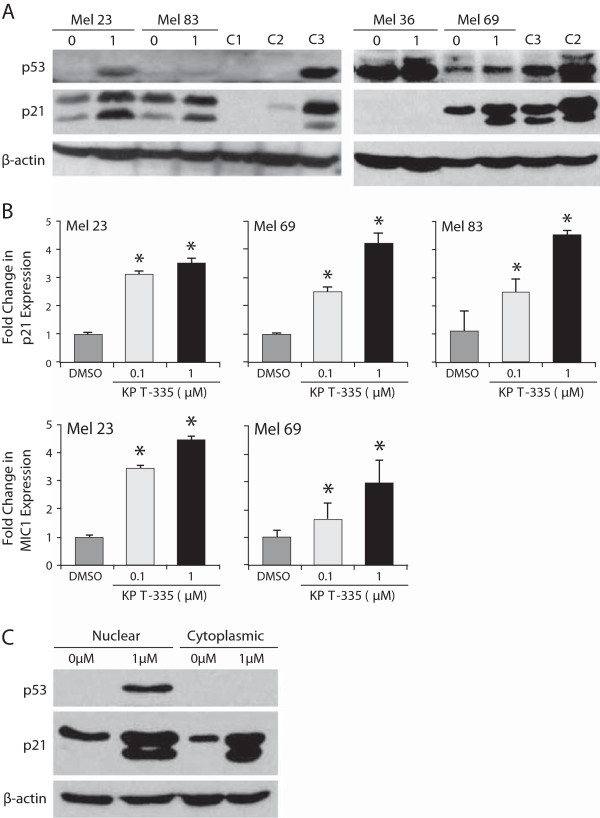
**Evaluation of p53 and p21 expression and localization in canine melanoma cell lines after KPT-335 treatment. A**. Canine melanoma cell lines were treated with DMSO or 1 μM KPT-335 for 24 hours prior to collection. Protein lysates were generated, separated by SDS-PAGE and Western blotting for p53, p21 and β-actin were performed. **B**. Canine melanoma cell lines were treated with DMSO or KPT-335 at 0.1 μM or 1 μM for 4 hours (Mel 69) or 24 hours (Mel 23 and Mel 83). RNA was collected and quantitative RT-PCR was performed for MIC-1 and relative expression of MIC-1 was significantly increased in Mel 23 and Mel 69 cells treated with 0.1 μM and 1 μM KPT-335 and MIC-1 was significantly increase in Mel 23 and Mel 69 cells treated with 0.1 μM and 1 μM (p < 0.05). **C**. Canine melanoma cell lines were treated with DMSO or 1 μM KPT-335 for 24 hours prior to collection. Nuclear and cytoplasmic protein lysates were generated and separated by SDS-PAGE and Western blotting for p53, p21 and β-actin was performed.

**Figure 6:**
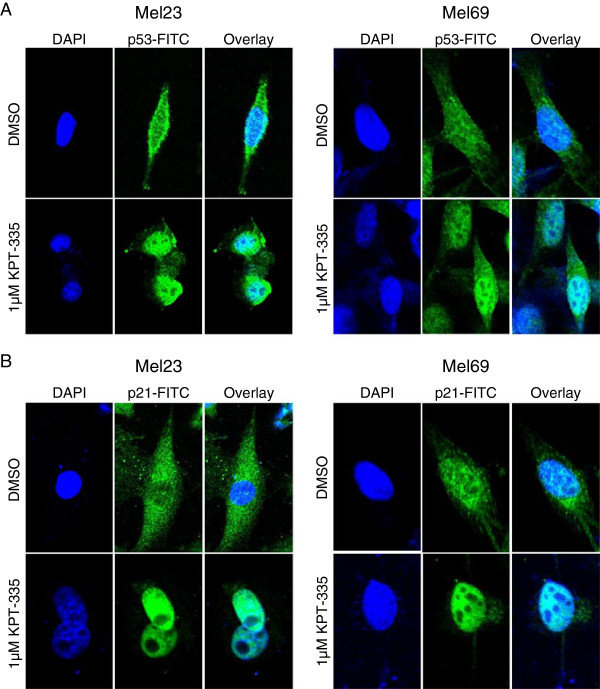
**Confocal microscopy to detect p21 and p53 cellular localization following KPT-335 treatment of melanoma cell lines.** The canine melanoma cell lines Mel 23 and Mel 69 were treated with DMSO or 1 μM KPT-335 for 24 hours. Cells were fixed and stained with ant-p21 **(A)** or anti-p53 **(B)** and then a secondary FITC conjugated antibody. Cells were then counterstained with DAPI to identify the nucleus. Images were obtained using an Olympus FV1000 Spectral confocal microscope and overlayed to assess protein localization.

Consistent with previous work, only 3 of the 4 lines expressed p53 protein [34]. KPT-335 treatment enhanced p53 expression in two of these lines after 24 hours of culture (Figure 5A). Quantitative RT-PCR showed that expression of the targets p21 and MIC-1 was significantly upregulated after canine melanoma cell lines were treated with DMSO or KPT-335 at 0.1 μM or 1 μM for 4 hours (Mel 69) or 24 hours (Mel 23 and Mel 83) (Figure 5B). Mel 69 was collected at an earlier time point because this cell line grew at a much faster rate than the other lines so overgrowth in the vehicle control group was a problem at later time points of collection. MIC-1 was only significantly increased in Mel 23 and Mel 69. Similarly, p21 was expressed in 3 of the 4 lines and exposure to KPT-335 increased its expression. Evaluation of nuclear versus cytoplasmic protein in treated cells revealed that with drug treatment both p53 and p21 expression were increased primarily in the nucleus, although levels were somewhat enhanced in the cytoplasm as well (Figure 5C). Lastly, confocal microscopy confirmed that p53 and p21 did demonstrate enhanced nuclear localization after KPT-335 treatment in Mel 23 and Mel 69 (Figure 6).

## Discussion

Unlike malignant melanoma in humans which occurs primarily in the skin and is typically associated with exposure to UV irradiation, malignant melanoma in dogs occurs primarily in the oral cavity and nail beds [1]. Approximately 60% of human cutaneous melanomas possess activating mutations in the cytoplasmic kinase BRAF whereas these mutations are not found in the canine disease [41,42]. In both dogs and people, standard chemotherapy agents do not have substantial activity in either the primary disease or in the setting of metastasis [1,3-7,43]. While there are significant differences that exist between the clinical presentation and molecular biology of human and canine malignant melanoma, two recent review articles demonstrated that the diseases do share similar histopathologic features as well as alterations in both AKT and MAPK signaling pathways, with *RAS* and *PTEN* mutations present in tumors from both species [42,44].

In human patients with melanomas that carry *BRAF* mutation the small molecule BRAF inhibitor vemurafenib (Zorafenib) has shown significant activity with response rates of approximately 50%. Unfortunately, these responses tend to be short-lived, and most patients relapse within 8–12 months [45,46]. More recently, monoclonal antibodies that block signaling of CTLA4 (ipilumimab, Yervoy) and PD1 (nivolumab) have induced durable clinical remissions in human patients with widely disseminated metastatic melanoma; when the two antibodies were combined, response rates were over 40% and in many instances, were durable [47,48].

There are currently no monoclonal antibodies that block CTLA4 or PD1 available for use in veterinary medicine, and while the chemotherapy agent carboplatin has demonstrated some activity against primary oral malignant melanoma in dogs, there is no definitive evidence that any chemotherapy agents work in the microscopic disease setting. Recently, the Oncept xenogeneic melanoma vaccine was approved for use in dogs with oral malignant melanoma in the adjuvant setting following control of the primary tumor/local disease [49]. However, many dogs still develop widespread metastatic disease necessitating the development of new therapeutic approaches.

Selective inhibitor of nuclear export (SINE) compounds that target the nuclear export protein XPO1 represent a potential target for therapeutic intervention in canine and human melanoma. Several studies have demonstrated that treatment of tumor cell lines with SINE compounds results in enhanced expression of p21 and p53, and redistribution of these proteins into the nucleus [26,28,29,31,32,45]. With respect to melanoma, both p53 and retinoblastoma protein (Rb) undergo increased nuclear export preventing them from engaging in active tumor suppression [50]. In canine melanoma p53 was shown to be excluded from the nucleus in 7 cell lines and 18 of 25 tumor samples [51]. The importance of p53 activity independent of mutation status has been demonstrated in human melanoma and therapies that increase the activity of this protein have shown promise [52-54]. Following treatment of human melanoma lines with a variety of SINE compounds (KPT-185, −251, −276, 330) in combination with a small molecule BRAF inhibitor, stabilization of p53 was shown to be at least partly responsible for induction of cell cycle arrest and apoptosis [45]. Furthermore, the combination of XPO1 and BRAF inhibition was found to be synergistic, also altering the expression of Rb and survivin [45].

Given the similarity of canine and human melanoma with respect to tumor suppressor dysregulation, we were interested in determining whether SINE compounds would exhibit biologic activity against canine melanoma cells. Several SINE compounds have been tested *in vitro* and found to have good activity at nanomolar concentrations against human prostate, renal, pancreatic, and breast cancer cell lines, as well as against a variety of cell lines representing hematopoietic malignancies (CLL, mantle cell lymphoma, NHL, AML) [25-28,30,31]. Currently, the SINE compound KPT-330 (selinexor) is undergoing evaluation in human clinical trials, while the SINE compound KPT-335 (verdinexor) has completed both phase 1 and 2 canine clinical trials. Significant activity against canine NHL was observed *in vitro* in the low nanomolar range against canine diffuse large B cell lymphoma (DLBCL) samples, as well as in a canine DLBCL cell line [33]. In the phase 1 setting, partial response to KPT-335 was observed in dogs with NHL, and unlike previously tested XPO1 inhibitors such as leptomycin B [55], KPT-335 exhibited good tolerability with anorexia and weight loss as the main clinical effects noted [33]. Based on these findings, we elected to evaluate the potential utility of this SINE compound in canine melanoma lines prior to clinical testing in dogs with melanoma.

In the current study we found that KPT-335 inhibited the proliferation of canine melanoma cell lines with IC_50_ concentrations ranging from 71–330 nM. These doses are biologically relevant and achievable based on pharmacokinetic data derived from studies in both healthy dogs and dogs enrolled in the phase 1 and 2 studies [33]. Furthermore, we demonstrated that KPT-335 induced apoptosis of the melanoma cell lines, although these effects were somewhat delayed, occurring at 72–96 hours after drug exposure. This delay in effect is concordant with prior data generated in human melanoma lines in which BCL-2 was not down regulated until 32 hours following exposure to SINE compounds [56]. Additionally, in human leukemia cell lines treated with SINE compounds, cycle arrest was noted at 24 hours but IC₅₀ values were best at 48 and 72 hours [45].

Although significant changes in proliferation or apoptosis were not found until 72–96 hours after drug exposure, the melanoma cell lines demonstrated changes in gene and protein expression at much earlier time points. This is likely a consequence of the manner in which tumor cells are disrupted. Inhibition of XPO1 results in a redistribution of nuclear proteins and consequently, an impact on gene transcription, largely due to restoration of tumor suppressor protein localization. This initiates a genome survey to determine whether sufficient critical non-repairable errors exist that necessitate induction of apoptosis. During this time, the cells may be able to compensate for changes in nuclear and cytoplasmic localization of key proteins for a period of time through such mechanisms as autophagy, until a critical point is reached and the process of apoptosis is initiated [57]. The mechanism of action of XPO1 inhibitors is thus substantially different from that associated with small molecules that block known “driver” proteins often constitutively active through mutation, translocation or overexpression. In these cases, blocking the function of the driver protein typically results in rapid cell death as the tumor cell has become reliant on that particular protein for survival.

As expected, treatment of melanoma lines with KPT-335 increased the protein expression of p53 and p21 and the mRNA of p21 and MIC-1. There also was enhanced nuclear translocation of p21 and p53. Interestingly, the Mel 36 line, previously reported to be p53 null [34], was found to have basal p53 protein expression that increased after drug exposure. The reason for this discrepancy is not known, although it is possible there have been alterations in gene expression profiles over time. The effects of XPO1 inhibition on expression of these tumor suppressor proteins have been well-documented in a variety of human tumor cell lines treated with SINE compounds and this is likely a significant contributor to loss of cell viability [26,28,29,31,32,45]. Prior studies with leptomycin B showed that prostate and neuroblastoma human tumor cell lines exhibited upregulation and activation of p53 that directly contributed to growth arrest and apoptosis [58,59]. In contrast, apoptosis after inhibition of XPO1 can occur regardless of p53 status in some tumor lines indicating that this is not the sole mechanism for cell death [58]. Human melanoma cell lines were shown to undergo apoptosis after SINE compound exposure regardless of p53 status and other mechanisms such as enhanced PUMA expression and decreased activation of NF-ĸB were found to be responsible [32]. These data are concordant with our studies as all melanoma lines treated underwent apoptosis independent of p53 status.

In addition to effects on transcription factors, our data demonstrate a direct effect of KPT-335 on its target protein, XPO1. Downregulation of XPO1 protein was observed in melanoma cell lines as early as 4 hours after drug exposure and expression was nearly completely eliminated by 24 hours of treatment. These findings are concordant with those generated in human myeloma cells treated with the KPT-185 and KPT-330 [32]. Similarly, human myeloma cells treated with the reversible XPO1 inhibitor CBS9106 exhibited downregulation of protein that was dependent on the ubiquitin/proteasome pathway [60]. While protein was decreased, messenger RNA for XPO1 was increased in the canine melanoma lines following KPT-335 exposure, a finding also observed in human cell lines treated with the SINE compounds [32]. This likely represents a compensatory mechanism associated with loss of functional XPO1 protein. In fact, upregulation of XPO1 message is now being used as a biomarker of target inhibition in ongoing human clinical trials of the SINE compound KPT-330 (selinexor) [40].

## Conclusion

In summary, KPT-335 (verdinexor), a novel orally bioavailable XPO1 inhibitor exhibits good *in vitro* single agent activity against canine malignant melanoma cell lines as evidenced by inhibition of proliferation and induction of apoptosis. KPT-335 downregulated XPO1 protein while upregulating XPO1 message, indicative of direct effects on the intended target. The biologic effects of KPT-335 were demonstrated when cells were treated with nanomolar concentrations of drug which have been shown to be biologically achievable in dogs following oral administration. These data lay the groundwork for future clinical evaluation of KPT-335 in dogs with malignant melanoma.

## Competing interests

KPT-335 was supplied by Karyopharm, Therapeutics. Yosef Landesman, Trinayan Kashyap, Dilara McCauley, Michael Kauffman and Sharon Shacham are employed by Karyopharm, Therapeutics, Inc.

## Authors’ contributions

MNB carried out experiments on melanoma cell lines and drafted the manuscript. MDB assisted with the experiments performed in this study. WK and YL assisted in experimental design. TK performed real time PCR for TSPs. DM, MK and SS assisted with project direction and edited the manuscript. CL conceived the study, assisted in experimental design and helped draft the manuscript. All authors read and approved the final manuscript.
